# Fabrication of Strontium Molybdate with Functionalized Carbon Nanotubes for Electrochemical Determination of Antipyretic Drug-Acetaminophen

**DOI:** 10.3390/ma17122887

**Published:** 2024-06-13

**Authors:** Dhanashri D. Khandagale, Sea-Fue Wang

**Affiliations:** Department of Materials and Mineral Resources Engineering, National Taipei University of Technology, Taipei 106, Taiwan; t111a09407@ntut.edu.tw

**Keywords:** screen-printed carbon electrode, acetaminophen, synergistic effect, SrMoO_4_@f-CNF, electrochemical sensor

## Abstract

In recent years, there has been a significant interest in the advancement of electrochemical sensing platforms to detect antipyretic drugs with high sensitivity and selectivity. The electrochemical determination of acetaminophen (PCT) was studied with strontium molybdate with a functionalized carbon nanotube (SrMoO_4_@f-CNF) nanocomposite. The SrMoO_4_@f-CNF nanocomposite was produced by a facial hydrothermal followed by sonochemical treatment, resulting in a significant enhancement in the PCT determination. The sonochemical process was applied to incorporate SrMoO_4_ nanoparticles over f-CNF, enabling a network-like structure. Moreover, the produced SrMoO_4_@f-CNF composite structural, morphological, and spectroscopic properties were confirmed with XRD, TEM, and XPS characterizations. The synergistic effect between SrMoO_4_ and f-CNF contributes to the lowering of the charge transfer resistance ($R_{ct}=85\;Ω\cdot {cm}^{2}$), a redox potential of $E_{pc}=0.15\;V$ and $E_{pa}=0.30\;V$ (vs. Ag/AgCl), and a significant limit of detection (1.2 nM) with a wide response range of 0.01–28.48 µM towards the PCT determination. The proposed SrMoO_4_@f-CNF sensor was studied with differential pulse voltammetry (DPV) and cyclic voltammetry (CV) techniques and demonstrated remarkable electrochemical properties with a good recovery range in real-sample analysis.

## 1. Introduction

Acetaminophen, often known as paracetamol (PCT), is a commonly used antipyretic and analgesic medicine in medical care. It cures fever, headaches, joint pain, sore throat, colds, flu, and post-surgical pain. Primarily, PCT is generally safe and has no significant negative effects when taken as prescribed [1]. However, the excessive use of PCT may produce toxic substances, which could cause hepatotoxicity and nephrotoxicity [2,3]. Moreover, the PCT introduced into aquatic environments through hospital wastewater and pharmaceutical waste pollutes the ecosystem by mixing with waterbodies [4]. It may lead to acute hepatic and liver failure through the accumulation of PCT in the liver and kidney. The safe level of PCT in the environment and for human health ranges from ng L^−1^ to µg L^−1^ [1,5], Traditional wastewater treatment plants (WWTPs) are unable to effectively mineralize drug molecules, resulting in disinfection byproducts that are more harmful than the original compounds [6,7]. Therefore, the quick and accurate monitoring of acetaminophen in the human body and environment is becoming more essential for public health and accurate treatment. Currently, several approaches have been reported for determining PCT, including UV spectrophotometry [8], spectrofluorimetric [9], high-performance liquid chromatography [10], and gas chromatography techniques [11], which can detect a trace amount of PCT. However, these techniques have some drawbacks such as expensive facilities, the need for additional solvents, and lengthy, time-consuming analysis. Recently, electrochemical techniques [12] have received great attention in the determination of environmental pollutants, relaying the redox current–potential response using screen-printed electrodes [13]. However, PCT consists of hydroxyl and acetamido functional groups that are electrochemically sensitive [1]. In the literature, researchers reported the electrochemical determination of PCT, although the sensitivity, storage stability, and real-time monitoring need to be optimized with the electrocatalyst.

Transition metals play a crucial role in electrochemical applications due to their exciting properties, such as significant conductivity, stability, and non-toxic and superior electrochemical properties [14,15,16,17]. Metal molybdate is a promising electrode material owing to its high electrical conductivity, eco-friendly, superior optical properties, electrochemical performance, and significant stability [18,19]. It has been studied in different fields, including catalysis, lithium-ion batteries, photocatalysis, electrochemical sensors, and so on [20]. Within them, SrMoO_4_ is a fascinating metal from the perspective of an electrochemical sensor due to its low cost, easy synthesis method, high yield, and earth abundance. Based on the literature the electrochemical performance of molybdates may be improved with the carbon matrix [21,22] (f-CNF, multiwall carbon nanotubes) [23]. Molybdate with f-CNF composite has been reported for an electrochemical determination study [24]. Over the past decades, different types of materials have been used as electrode modifiers to enhance PCT detection: Palladium-doped α-MnO_2_ nanorods on graphene [25], a MoS_2_–TiO_2_/reduced graphene oxide nanocomposite [26], Hf-doped tungsten oxide nanorods [27], mesoporous graphitic carbon nitride/black phosphorus/gold nanoparticles [28], and black phosphorus nanosheets [29]. The novelty of this research work lies in using SrMoO_4_ with f-CNF-decorated screen-printed electrodes to determine PCT. Integrating SrMoO_4_ with f-CNF further increases the electrochemically catalytic activity of the composite. The active sites of f-CNF enhance the active surface area and fast electron transfer, and SrMoO_4_ offers promising electrocatalytic properties. The electrochemical behavior of PCT, especially when bound to metal sites as a precursor, is considerably enhanced when surrounded by a highly porous carbon matrix. F-CNF has a unique stacking arrangement in morphology which results in an abundance of edge sites on the periphery of f-CNF [30,31,32]. Moreover, the proposed SrMoO_4_@f-CNF composite substantially decreases electrode fouling, improving electrochemical performance, which makes it a significant tool for PCT monitoring. 

The current study represents the modification of screen-printed electrodes with an equal weight ratio of SrMoO_4_ and CNF for PCT electrochemical detection. The prepared electrode properties were studied with an active surface area, and the reaction pathways were evaluated using CV and DPV techniques. Additionally, the successful formation of SrMoO_4_@f-CNF composite was studied with structural and morphological analysis. To assure superior control of PCT, the detection limits, recovery, sensitivity, interference, and real-time determination in tablet and water samples were studied.

## 2. Experimental

### 2.1. Chemicals and Reagents

The chemicals strontium nitrate (Sr(NO_3_)_2_ ≥99.5%), sodium molybdate (Na_2_MoO_4_·H_2_O ≥99.0%), urea (CO(NH_2_)_2_ ≥98.0%), hydrochloric acid (HCl ≥37%), sodium hydroxide (NaOH ≥97.0%), disodium hydrogen phosphate (Na_2_HPO_4_ ≥99.0%), sodium dihydrogen phosphate (NaH_2_PO_4_ ≥99.99%), acetaminophen ($C_{8}H_{9}{NO}_{2}\geq 98.0\%),$ potassium ferri-ferrocyanide (([Fe(CN)_6_]^3−,4−^) ≥99.9%), and potassium chloride (KCl ≥99.5%) were all purchased from Sigma-Aldrich, Shanghai, China, Showa, Alfa Aesar chemicals and were cast-off further without any purification process. All the other chemicals were of analytical grade. Deionized (DI) water was obtained from a Millipore water purification system (Milli-Q, specific resistivity > 18 MΩcm, S.A.; Molsheim, France), which was used throughout the experimental study. The electrochemical studies were conducted with 0.1 M phosphate buffer solution (PBS) (pH-6.0) as the supporting electrolyte.

### 2.2. Electrochemical Measurement

CV and DPV were performed on a CHI1211C electrochemical workstation controlled with three three-electrode systems to test the electrochemical performance. The electrode system consisted of a platinum wire as a counter electrode, a Ag/AgCl electrode as a reference electrode, and SPCE as a working electrode. For the DPV and CV measurements, a 50 mV scan rate and a potential range of 0 to 0.8 V were used.

### 2.3. Preparation of Functionalized Carbon Nanotubes

To activate a pristine CNF edge site, an acid treatment approach was used. In brief, concentrated nitric acid and sulfuric acid were prepared in a 1:3 volumetric ratio for this approach. The addition of CNF to the acidic mixture causes it to disperse into a dense solution that must be constantly stirred at 60 °C for one hour at 400 rotations per minute (rpm) at room temperature. Furthermore, to remove the acidic mixture, the final solution was centrifuged many times using DI water to maintain the neutral potential of hydrogen (pH) of the solution. The centrifuged slurry was left in an oven for the entire night to dry. With the addition of oxygen functional groups to CNF, the functionalization results in increased electrochemical activity and solubility [33,34]. Finally, the produced black powder was referred to as f-CNF and studied for further characterization and electrochemical studies.

### 2.4. Synthesis of SrMoO_4_

**Scheme 1:**
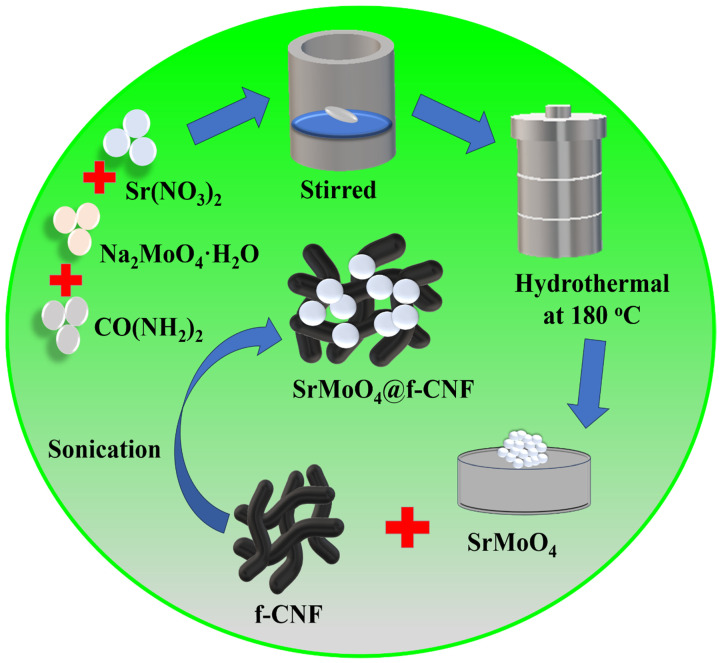
Schematic representation for the synthesis of SrMoO_4_@f-CNF.

An easy hydrothermal approach was applied for SrMoO_4_ synthesis using Na_2_MoO_4_·H_2_O and Sr(NO_3_)_2_ as precursors and CO(NH_2_)_2_ as a reaction rate promotor. In detail, 70 mL was placed in a beaker, and an equal molar ratio of Na_2_MoO_4_·H_2_O and Sr(NO_3_)_2_ was added under stirring at 800 rpm; after obtaining a homogeneous mixture, urea was poured into the same solution and stirring was performed for a further 5 min. Then, the homogenous solution was poured into a Teflon layer autoclave and kept hydrothermal at 180 °C for 24 h (Scheme 1). After completing the reaction, the obtained solution was washed several times with DI water and ethanol to collect the residue further; it was kept for drying at 80 °C overnight, and a white color powder named as SrMoO_4_ was obtained.

### 2.5. Synthesis of SrMoO_4_@f-CNF

Hydrothermally synthesized SrMoO_4_ was followed by the incorporation of the f-CNF network using the sonication method. This approach maintained the original morphological and structural features of the SrMoO_4_ material. In detail, first a homogeneous mixture was achieved by dispersing equal-weight ratios of SrMoO_4_ and f-CNF in 10 mL of deionized water. Afterwards, the produced slurry was stirred for 20 min at 400 rpm and then kept for sonication for 1 h at 100 W and 50 kHz; the temperature was maintained at room temperature. (Scheme 1) Then, the resulting homogeneous slurry was dried at 60 °C overnight, and the obtained product was ground with the help of a pestle and mortar to collect the powder form. Furthermore, the collected powder was named SrMoO_4_@f-CNF, which was characterized and studied for its electrochemical properties.

### 2.6. Preparation of Electrode

The electrochemical study for PCT was performed with SPCE as a working electrode. An equal weight ratio of SrMoO_4_@f-CNF nanocomposite was mixed with a pestle and mortar and then poured into 1 mL of DI water and kept for 1 h. Then, sonication was performed to obtain a thick slurry. The liquid-like paste of SrMoO_4_@f-CNF nanocomposite was subsequently drop-cast onto the SPCE working electrode surface. The drop-cast SPCE working electrode was dried in 60 °C oven for 10 min. Furthermore, this fabricated SPCE was used for the electrochemical measurement of the PCT determination. The main benefit of using SPCE as a working electrode surface is that it provides significant sensitivity and significant reproducibility results and is more resistant to mechanical fracture from bending. Furthermore, fabricated nanocomposite on the SPCE surface does not leach out when it contacts with electrolyte over time.

### 2.7. Real-Sample Preparation

The real-sample study of the electrochemical sensor was performed to determine PCT in river water and tablet samples. Sample preparations followed, as described below:

- River water sample: The river water was collected from the Taipei River, Taiwan. Then, an equal amount of collected river water and pH-6.0 was put into a centrifuge tube and centrifuged for 10 min at 4000 rotations per minute (rpm) [35]. The obtained supernatant was diluted with pH-6.0 and used to analyze the real sample.
- Tablet sample: The commercially available 500 mg tablet was purchased from a nearby medical store, in Taipei. Then, the table was crushed into fine powder with the help of a pestle and mortar and diluted with 100 mL of pH-6.0 and centrifuged for 10 min at 4000 rpm [36]. Then the supernatant was collected from the obtained solution and further used to determine real-sample analysis.

## 3. Results and Discussion

### 3.1. XRD Analysis

**Figure 1:**
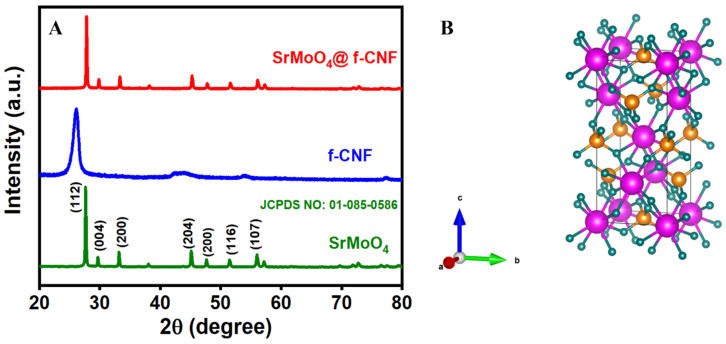
(**A**) XRD pattern for f-CNF, SrMoO_4_, and SrMoO_4_@f-CNF composite, (**B**) crystal structure of SrMoO_4_.

The XRD examination revealed the crystal arrangement and composition of the prepared materials. The diffraction patterns for SrMoO_4_, f-CNF, and SrMoO_4_@f-CNF are plotted in Figure 1. It disclosed a SrMoO_4_ tetragonal structure formation with strong agreement with JCPDS 01-085-0586, combined with lattice constants of a = 5.394 Å and c = 12.02 Å and a I41/a(88) space group number, respectively [37]. Figure 1B depicts the crystal structure of SrMoO_4_, where the synthesized nanoparticles show a tetragonal crystal system. The prominent peaks suggest good sample crystallinity, and no additional peaks were observed, confirming SrMoO_4_ with high purity. Furthermore, the composite synthesis with f-CNFs has a low peak that is closely related to a high-intensity, abrupt peak of SrMoO_4_ nanoparticles at 26.30° (002). The X-ray diffractogram demonstrated the successful formation of high-purity SrMoO_4_@f-CNF composites.

### 3.2. TEM Analysis

**Figure 2:**
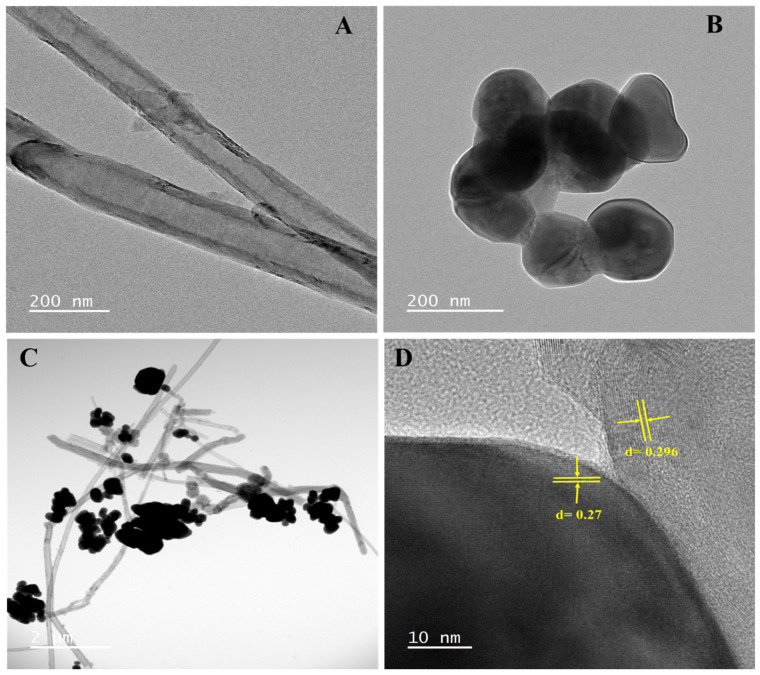
TEM micrographs of (**A**) f-CNF, (**B**) SrMoO_4_, (**C**) SrMoO_4_@f-CNF, and (**D**) lattice fringe.

The morphological studies were performed with TEM analysis of the prepared materials. TEM analysis gives clearer information about the f-CNF, SrMoO_4,_ and SrMoO_4_@f-CNF composite formation and its morphology. Figure 2A–C depict the micrographs of the f-CNF, SrMoO_4,_ and SrMoO_4_@f-CNF composites, respectively, where the results reveal the well formation of the native materials and the composite through its morphology. SrMoO_4_ formed in a sphere shape, and the SrMoO_4_@f-CNF composite depicts the well distribution of f-CNF over the SrMoO_4_ nanoparticles. The obtained morphology improves the electrochemical sensing properties towards PCT detection. As denoted in Figure 2D, the lattice fringe for SrMoO_4_ is d = 0.27, and the graphitic lattice fringe d = 0.296 corresponds to f-CNF, representing the successful formation of prepared materials [38]. Moreover, Figure S1 shows the TEM image of the SrMoO_4_@f-CNF composite, the elemental color mapping, and EDX; the results reveal the homogeneous presence of Sr, Mo, O, and C elements.

### 3.3. XPS Analysis

**Figure 3:**
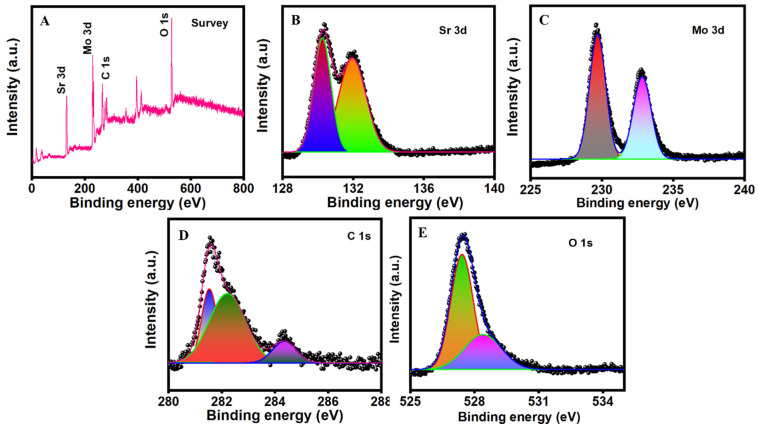
(**A**) XPS survey, (**B**) Sr 3d, (**C**) Mo 3d, (**D**) C1s, (**E**) O 1s. (Note-line+symbol shows raw data and filled color shows fit data).

To examine the surface chemical and valence state of the produced materials, an XPS spectrum was examined; the results are displayed in Figure 3A. The Sr 3d XPS spectra shown in Figure 3B examined two distinct peaks at 130.6 eV and 132.0 eV, which corresponded to Sr^2+^ 3d_5/2_ and Sr^2+^ 3d_3/2_, respectively, and disclosed the Sr ion with the divalent oxidation state [39]. Similarly, the Mo 3d high-resolution XPS spectra shown in Figure 3C examined the two peaks at 229.7 and 232.8 eV, which matched to the Mo^6+^ 3d_5/2_ and Mo^6+^ 3d_3/2_ and pointed out that the Mo ion has an hexavalent oxidation state [40]. The high-resolution O 1s spectra, shown in Figure 3D, were indicated by the focusing oxygen energy [38] peak at 527.4 and 528.6 eV and were associated with the O^2−^ lattice oxygen. The carbon energy peak at 284.6 eV, which represents the C=C bond, was identified in the XPS spectra of C1s, as shown in Figure 3E. The peak at 282.4 eV may be related to C−C bonds. Additionally, the presence of a C−O bond in the XPS spectra indicated the formation of chemical bonding between SrMoO_4_ and f-CNF.

### 3.4. Electrochemical Study

#### 3.4.1. EIS Study

**Figure 4:**
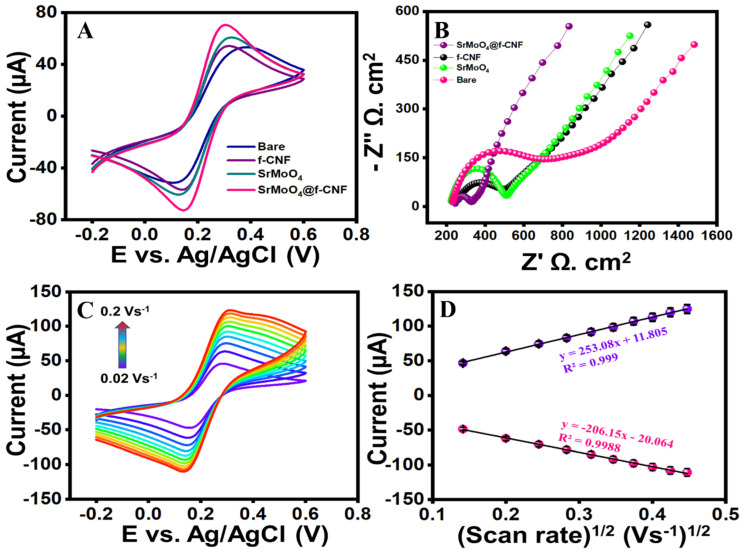
(**A**) EIS measurements, (**B**) CV curves of bare and functionalized electrodes in redox ferricyanide system, (**C**) different scan rates for SrMoO_4_@f-CNF electrode in redox ferricyanide system, and corresponding (**D**) linear graph.

The EIS study was performed for the analysis of electron transfer impedance (Rct) and the behavior of the electrode–electrolyte interface. The experiment was performed in a redox ferricyanide system (5 mM) to study the electrode kinetic of the modified and unmodified electrodes. As depicted in Figure 4B, the EIS results show the better conductivity of the SrMoO_4_@f-CNF electrode than the f-CNF, SrMoO_4_, and bare electrode. Here, the synergism of the SrMoO_4_@f-CNF composite gives a lower resistance value ${(R}_{ct}=85\;Ω\cdot {cm}^{2}$) and enhanced electrocatalytic properties. The two control materials (f-CNF, SrMoO_4_) and the bare electrode show higher semicircle values than the SrMoO_4_@f-CNF composite. The diameter of the semicircle corresponds to the charge transfer barrier at the electrode surface. A higher diameter of the semicircle in the spectrum indicates high resistance to interfacial electron transfer.

#### 3.4.2. CV Study

CV analysis was used to study the conductivity of different modified and unmodified electrodes in the redox ferricyanide system. In addition, ferricyanide is a typically used redox system in electrochemical studies and undergoes reversible redox reactions without significant adsorption onto the electrode surface [41]. It can easily reach the electrode contact and take part in redox reactions, resulting in the CV curve shown in Figure 4A. The CV curves for f-CNF, SrMoO_4,_ and the SrMoO_4_@f-CNF electrode; the results demonstrate improved current response for the SrMoO_4_@f-CNF electrode at a 0.3 V potential and low peak-to-peak separation. The synergistic effect between SrMoO_4_ and f-CNF contributes to enhanced electrocatalytic properties. Furthermore, to study the electrode kinetics a scan rate was performed in the redox ferricyanide system in the 20 mV to 200 mV range. Figure 4C demonstrates the scan rate results, and Figure 4D shows a linear graph for the scan rate values of the oxidation peak, where the calculated linear plot of the square of the scan rate is R^2^ = 0.9990; this value is near to one, which shows the significant linear fit of the scan rate. Here, the calculated R^2^ value of the square root of the scan rate shows the surface control process on the electrode surface [35].

### 3.5. Electrochemical Analysis of PCT on SrMoO_4_@f-CNF Electrode

#### 3.5.1. Different Film

**Figure 5:**
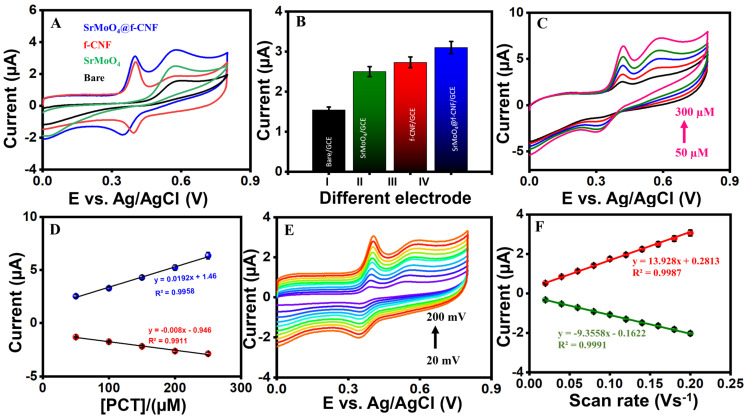
CV readings of (**A**) various modified and unmodified electrodes, (**B**) corresponding bar diagram, (**C**) various concentrations on SrMoO_4_@f-CNF electrode, (**D**) corresponding linear plot, (**E**) various scan rates from 20 to 200 mV, (**F**) linear graph of scan rate.

The CV study was conducted at a 50 mV scan rate using 100 M of PCT in pH-6.0 to characterize the electrochemical properties of different modified and unmodified electrodes. Figure 5A shows the CV readings of the f-CNF, SrMoO_4_, and SrMoO_4_@f-CNF electrodes respectively, where the SrMoO_4_@f-CNF electrodes show enhanced current response compared to the other modified and unmodified electrodes, as depicted in the calibration plot in Figure 5B. The sluggish charge transfer kinetics at the electrode–electrolyte interface causes the weakly addressed redox peak at the bare electrode. Moreover, all of the fabricated electrodes show enhanced current response peaks towards PCT detection. PCT is a well-known antipyretic drug and exhibits adsorptive behavior at certain electrode surfaces [7]. When PCT is added to the pH-6 electrolyte, it can adsorb onto the surface of the SrMoO_4_@f-CNF electrode. This adsorption process is likely driven by interactions between the PCT molecules and the electrode surface. Here, the SrMoO_4_@f-CNF composite reveals improved electrochemical properties towards the PCT determination because of the synergism between the constituent materials.

#### 3.5.2. Determination of PCT on SrMoO_4_@f-CNF Electrode

**Scheme 2:**
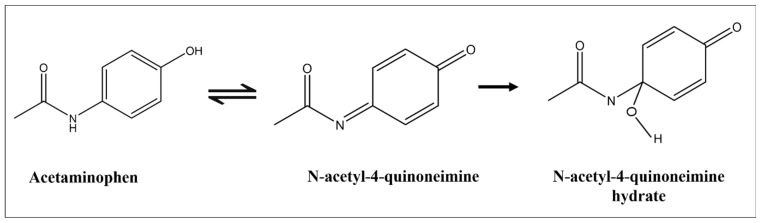
Plausible electrochemical reaction of PCT.

The CV performed for PCT determination at the SrMoO_4_@f-CNF electrode at the 50 mV scan rate is depicted in Figure 5A When the electrode potential is shifted in the positive direction while undergoing the two-electron oxidation of PCT, an anodic (oxidizing) current is detected. N-acetyl-4-quinoneimine causes quick hydrate formation. Due to the lack of electrochemical activity, the hydrate cannot be converted back into PCT. This means that the process cannot be interrupted by shifting the peak potential of the electrode in the opposite direction of its starting point. This type of reaction is referred to as irreversible in an electrochemical system. The PCT response on the electrode is as depicted in the following Scheme 2:

#### 3.5.3. Influence of Various pH Values

The different pH mediums of the electrolytes significantly influence the electrochemical behavior of modified electrodes [41]. Furthermore, to study the impact of different pH values from 3.0 to 11.0, CV was performed at a 50 mV scan rate and a 100 µL concentration of PCT. Figure S2A demonstrates the results based on different mediums of the pH range, where the pH-6.0 shows an enhanced current response for the PCT determination. Furthermore, there is a shift in peak potential as the pH value varies; the acid medium responds at a higher potential, while the basic medium responds at a lower potential. From the obtained results, the pH-6.0 electrolyte is appropriate because it provides the best resolution and peak shape for the three selected analytes.

#### 3.5.4. Influence of Various Catalyst Loading Amounts

To study the influence of the different micro-liter drop-casting of SrMoO_4_@f-CNF, a composite material CV was performed in pH-6.0. As depicted in Figure S2B, the results illustrate the noticeable increase in current response as the catalyst loading increases at 3 mg/µL, 4 mg/µL, and 5 mg/µL; after 5 mg/µL, there was a sudden drop in the current response. This decrease in current response for 6 mg/µL may be due to the blockage of electron transfer at a high loading amount of the SrMoO_4_@f-CNF composite [33]. Hence, in the result, the 5 mg/µL loading catalyst amount was fixed for further electrochemical experiments.

#### 3.5.5. Analysis of Different Concentrations of PCT and Scan Rate Analysis

Here, Figure 5C shows the effect of different concentrations of PCT at the electrode. The electrode was investigated for different concentrations ranging from 50 µM to 250 µM, and the results demonstrate that as the concentration increases, so does the current response. Figure 5D shows the linear plot for different concentrations of PCT versus current response; the obtained linear regression equation for oxidation y=13.928x+0.2813 is calculated at R^2^ = 0.9987, which is near to one and shows a remarkable property. To further study the impact of the scan rate at the electrode from 20 mV to 200 mV, a PCT concentration of 100 µL was used. Figure 5E depicts successive rises in current response as the scan rate value increases; in Figure 5F, the corresponding linear graph of the scan rate versus the current shows a significant R^2^ value.

#### 3.5.6. Analysis of DPV Study

**Figure 6:**
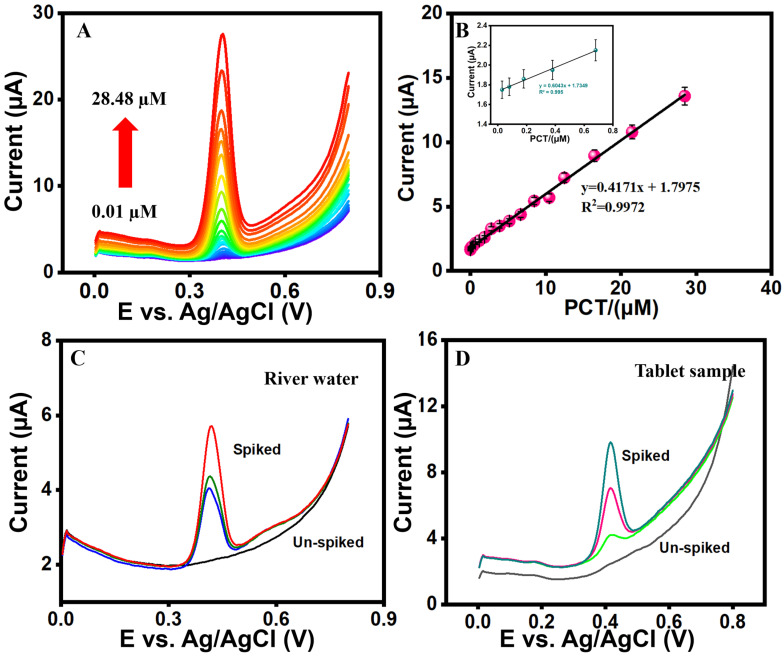
(**A**) DPV reading for PCT determination of SrMoO4@f-CNF electrode, (**B**) corresponding linear calibration plot; real-sample analysis with (**C**) river water, (**D**) tablet sample.

The DPV approach is more effective in electrochemical determination studies. Here, the SrMoO_4_@f-CNF electrode is employed to measure PCT concentration in the pH-6.0 electrolyte. Figure 6A depicts the DPV reading of the SrMoO_4_@f-CNF electrode at various PCT concentrations and indicates an enhancement in current response with an increase in PCT concentrations. Figure 6B shows a linear graph of DPV readings from 0.01 µM to 28.48 µM with an R^2^ value; the calculated limit of detection (LOD) value is 1.2 nM, which was obtained from the 0.3 µM to 0.68 µM concentration and used the following equation [37]:

(1) $$LOD=3\times \frac{SD}{m}$$

where SD is the standard deviation of the blank reading (the number is three), and m is analytical sensitivity.

The obtained LOD value was compared with previously reported research studies, and the results are given in Table S1. From this observation, the proposed electrode depicts the potential sensing properties of PCT’s electrochemical detection. The electrochemical sensor developed in this study exhibits remarkable sensitivity, detecting analytes at concentrations significantly lower than those reported in the recent literature. The linear response range is broader, allowing accurate quantification over a wider spectrum of concentrations, which enhances its practical applicability. It can be seen that the lowermost LOD has good sensitivity at 8.39 μA μM^−1^ cm^−2^, and a wide linear range is feasible at SrMoO_4_@f-CNF electrode compared to the previously reported works.

### 3.6. Precision, and Accuracy Measurements

#### 3.6.1. Cyclic Stability

The CV technique was performed to study the cyclic stability of PCT determination using the SrMoO_4_@f-CNF electrode in pH-6.0. Figure S2C shows the 40-segment stability, where the first cycle current is 5.26 µA and the last cycle current response is 4.68 µA. These results demonstrate a negligible drop in the current response (0.58 µA), which suggests that the proposed electrode has good potential for PCT determination.

#### 3.6.2. Selectivity Study

The interference analysis of the SrMoO_4_@f-CNF-modified electrode was studied with a DPV approach in pH-6.0. The different analytes were employed to study the selectivity towards PCT. This study aims to determine the electrode’s reliability in selective PCT detection, especially with organic compounds and metal ions, which can lead to the pollution of water systems by pharmaceutical waste. Figure S2D shows that the SrMoO_4_@f-CNF-modified electrode has remarkable selectivity for PCT, with negligible influence from the other investigated analytes. Firstly, the solution of PCT was spiked with a two-fold amount of interferons, and there was no current response increase with the emergence of interferon. Therefore, the proposed electrode has the potential for significant PCT determination. These results are attributable to the remarkable electrochemical properties of the SrMoO_4_@f-CNF composite for the PCT determination. Therefore, the SrMoO_4_@f-CNF-modified electrode could be the appropriate choice for PCT electrochemical detection.

#### 3.6.3. Reproducibility

The reproducibility analysis of the SrMoO_4_@f-CN-modified electrode was performed with a CV technique in a pH-6.0 electrolyte. Figure S2E depicts six measurements on different SPCEs modified with SrMoO_4_@f-CNF towards the PCT determination. The results demonstrate negligible difference in the current response, and the calculated relative standard deviation value is 1.8%. Hence, the proposed analysis technique and electrode reveal significant precision towards PCT detection.

#### 3.6.4. Analysis of Practicability

PCT offers the potential to address human health and aquatic environmental pollutants and is highly essential for quantitative analysis, to determine the concentration of PCT within different samples, including water and tablet samples. The DPV was used to perform real-time PCT analysis using the SrMoO_4_@f-CNF-modified electrode in pH-6.0 as an electrolyte. The prepared samples (without PCT) were studied with DPV readings, and there was no current response. Then, the known concentration of PCT was spiked into both samples and diluted with pH-6.0, and the DPV analysis showed the detection of PCT using the SrMoO_4_@f-CNF-modified electrode. As depicted in Figure 6C,D, each addition of PCT shows a gradual increase in the current response. Hence, the proposed electrode has significant potential in real-time analysis.

## 4. Conclusions

In summary, the SrMoO_4_@f-CNF composite was successfully developed with a simple hydrothermal followed by a sonication approach and characterized with structural and morphological analysis. The proposed electrochemical sensor has excellent electrocatalytic properties for PCT determination. Interestingly, the electrochemical sensor exhibited a significant linear range of 0.01–28.48 μM. The practical ability of the SrMoO_4_@f-CNF-modified electrochemical sensor was successfully applied in real-sample analysis. Therefore, the electrochemical study gives a significant outcome with a broad linear detection range, high selectivity, and considerable sensitivity of the proposed electrode. The calculated LOD value for the PCT detection is 1.2 nM, which is a significant value. Hence, the reported SrMoO_4_@f-CNF electrode may be a promising tool for PCT determination.

## Data Availability

The original contributions presented in the study are included in the article/supplementary material, further inquiries can be directed to the corresponding author.

## Supplementary Materials

The following supporting information can be downloaded at: https://www.mdpi.com/article/10.3390/ma17122887/s1, Figure S1: (A–F) TEM image and elemental color mapping of SrMoO_4_@f-CNF composite, (F) EDX; Figure S2: (A) CV curves for SrMoO_4_@f-CNF electrode at various pH medium towards 100 μM PCT, (B) different catalyst loading amounts of SrMoO_4_@f-CNF (C) cycle stability, (D) selectivity study, (E) reproducibility; Table S1: Analytical performance of different sensors for PCT detection; Table S2: Recovery study of spiked PCT in river water and tablet samples (n = 3). References [25,26,27,28,29,42,43,44,45] are cited in the supplementary materials.
