# Supplementary material for: Fabrication of Strontium Molybdate with Functionalized Carbon Nanotubes for Electrochemical Determination of Antipyretic Drug-Acetaminophen
Source: Materials (Basel). 2024 Jun 13;17(12):2887. doi: 10.3390/ma17122887 (PMC11204459; doi:10.3390/ma17122887)
Supplement: Supplementary file 1 [file materials-17-02887-s001.zip › materials-3012077-supplementary.pdf]

Supplementary Materials

Fabrication of Strontium Molybdate with Functionalized Carbon Nanotubes for Electrochemical Determination of Antipyretic Drug-Acetaminophen

Dhanashri D. Khandagale and Sea-Fue Wang *

Department of Materials and Mineral Resources Engineering, National Taipei University of Technology, Taipei 106, Taiwan; t111a09407@ntut.edu.tw

* Correspondence: sfwang@ntut.edu.tw

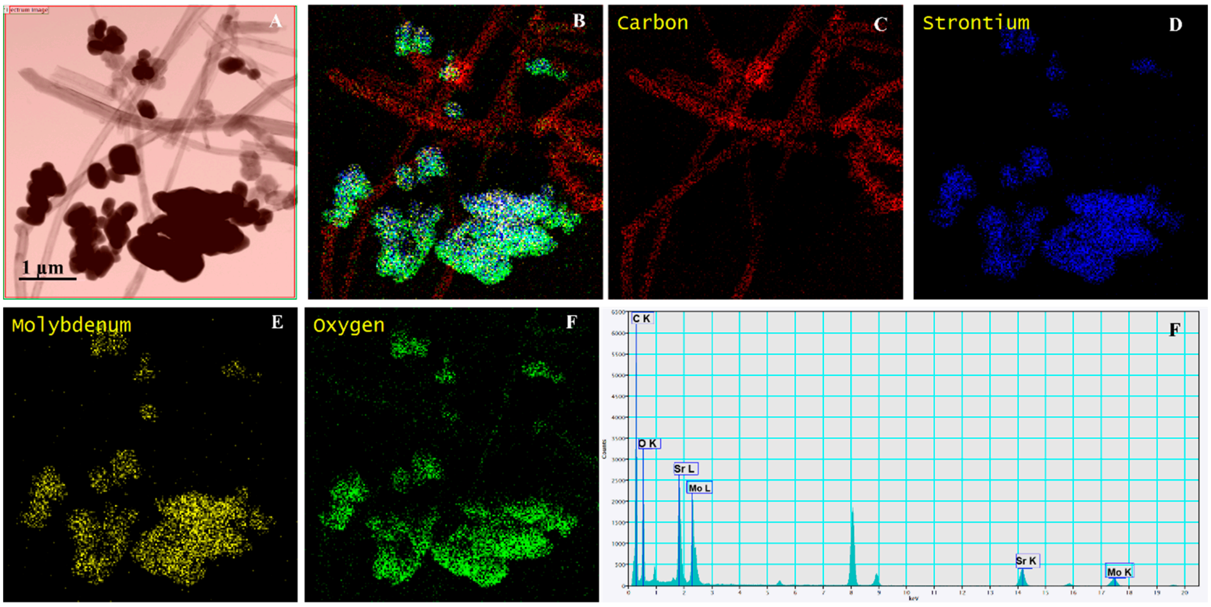

Figure S1. (A-F) TEM image and elemental color mapping of $SrMoO_4$@f-CNF composite, (F) EDX.

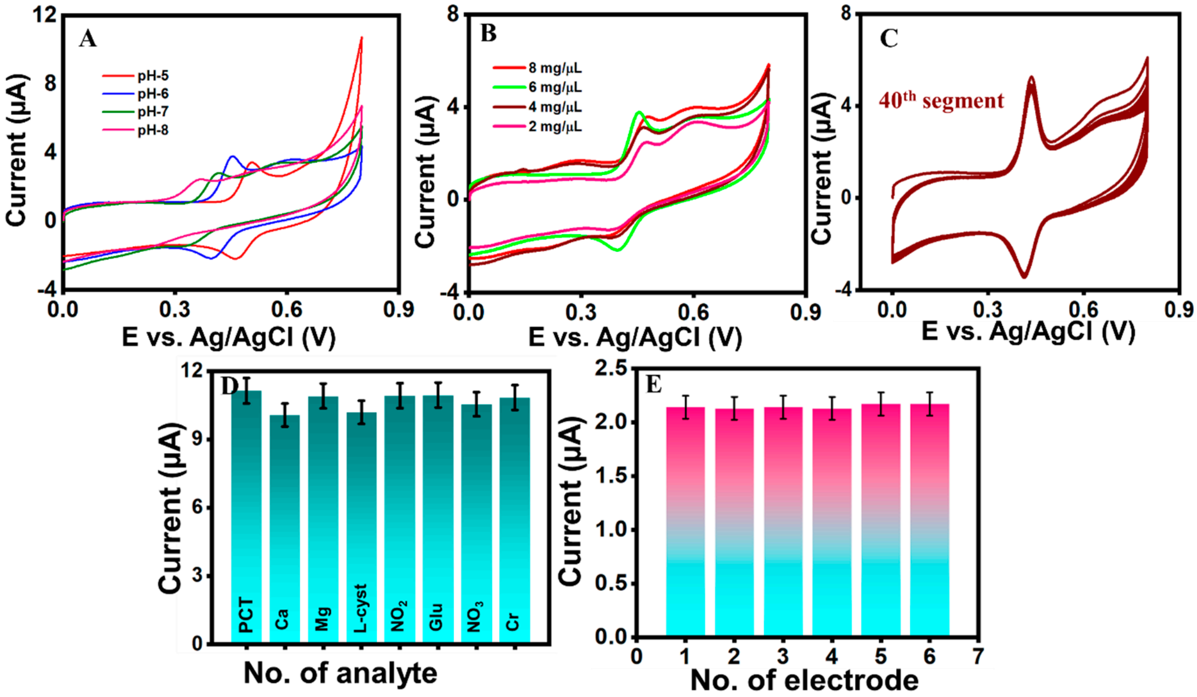

Figure S2. (A) CV curves for $SrMoO_4$@f-CNF electrode at various pH medium towards 100 μM PCT, (B) different catalyst loading amounts of $SrMoO_4$@f-CNF (C) cycle stability, (D) selectivity study, (E) reproducibility.

Table S1. Analytical performance of different sensors for PCT detection.

Electrode materials	Sensitivity ($\mu A\ \mu M^{-1}\ cm^{-2}$)	Linear range (µM)	LOD(µM)	Ref
N-Gr/PEDOT-MeSH & ($g-C_3N_4$/PEDOT-MeSH)	-	2 to 1680 & 0.4 to 1280	1.42 & 1.0	[1]
PTTPm/GCE	-	0.16 – 1300	0.06	[2]
$ZnO/ZnNi_2O_4$@porous carbon	18.40	48.5 − 130	0.0159	[3]
Pd@α-MnO_2/G	0.08	0.1 – 375	0.059	[4]
MoS2–TiO_2/rGO/SPE	0.44	0.1- 125	0.046	[5]
Hf.Wo_3/CPE	-	-	0.0128	[6]
mpg-CN/BP-Au	-	0.3–120	0.0425	[7]
BP NSs/GCE	-	0.01 – 2.4	0.00097	[8]
Cu-MOF/HNTs/rGO	-	0.5–250	0.15	[9]
$SrMoO_4$@f-CNF	8.39	0.01-28.48	0.0012	This work

Table S2. Recovery study of spiked PCT in river water and tablet samples (n = 3).

Real samples	Added (µM)	Found (µM)	Recovery (%)
River water sample	0	-	-
	5	4.83	96.6
	10	9.82	98.2

	15	14.85	99.0
Tablet sample	0	-	-
	5	4.87	97.4
	10	9.93	99.3
	15	14.95	99.6

Reference:

(1) Yan, Y.; Jamal, R.; Yu, Z.; Zhang, R.; Zhang, W.; Ge, Y.; Liu, Y.; Abdiryim, T. Composites of Thiol-Grafted PEDOT with N-Doped Graphene or Graphitic Carbon Nitride as an Electrochemical Sensor for the Detection of Paracetamol. *J Mater Sci* **2020**, *55* (13), 5571–5586. https://doi.org/10.1007/s10853-020-04351-w.

(2) Jamal, R.; Liu, Y.; Abdurexit, A.; Sawut, N.; Yan, Y.; Ali, A.; Abdiryim, T. Electrochemical Sensor for Detection of Paracetamol Based on Pendent Nitrogen Heterocyclic Ring-Functionalized Polyterthiophene Derivatives. *ChemistrySelect* **2021**, *6* (18), 4473–4481. https://doi.org/10.1002/slct.202100065.

(3) Luo, Y.; Yang, Y.; Wang, L.; Wang, L.; Chen, S. An Ultrafine ZnO/ZnNi2O4@porous Carbon@covalent-Organic Framework for Electrochemical Detection of Paracetamol and Tert-Butyl Hydroquinone. *J Alloys Compd* **2022**, *906*, 164369. https://doi.org/10.1016/j.jallcom.2022.164369.

(4) Kader Mohiuddin, A.; Shamsuddin Ahmed, M.; Jeon, S. Palladium Doped α-MnO2 Nanorods on Graphene as an Electrochemical Sensor for Simultaneous Determination of Dopamine and Paracetamol. *Appl Surf Sci* **2022**, *578*, 152090. https://doi.org/10.1016/j.apsusc.2021.152090.

(5) Demir, N.; Atacan, K.; Ozmen, M.; Bas, S. Z. Design of a New Electrochemical Sensing System Based on MoS_2 –TiO_2 /Reduced Graphene Oxide Nanocomposite for the

Detection of Paracetamol. *New Journal of Chemistry* **2020**, *44* (27), 11759–11767. https://doi.org/10.1039/D0NJ02298E.

(6) Shanbhag, M. M.; Shetti, N. P.; Kalanur, S. S.; Pollet, B. G.; Upadhyaya, K. P.; Ayachit, N. H.; Aminabhavi, T. M. Hf-Doped Tungsten Oxide Nanorods as Electrode Materials for Electrochemical Detection of Paracetamol and Salbutamol. *ACS Appl Nano Mater* **2022**, *5* (1), 1263–1275. https://doi.org/10.1021/acsanm.1c03853.

(7) Yanalak, G.; Doganay, F.; Eroglu, Z.; Kucukkececi, H.; Aslan, E.; Ozmen, M.; Bas, S. Z.; Metin, O.; Hatay Patir, I. Ternary Nanocomposites of Mesoporous Graphitic Carbon Nitride/Black Phosphorus/Gold Nanoparticles (Mpg-CN/BP-Au) for Photocatalytic Hydrogen Evolution and Electrochemical Sensing of Paracetamol. *Appl Surf Sci* **2021**, *557*, 149755. https://doi.org/10.1016/j.apsusc.2021.149755.

(8) Huang, J.; Qiu, Z.; Yang, H.; Chen, C.; Li, Y. Highly Selective Simultaneous Determination of Isoniazid and Acetaminophen Using Black Phosphorus Nanosheets Electrochemical Sensor. *Electrochim Acta* **2022**, *426*, 140775. https://doi.org/10.1016/j.electacta.2022.140775.

(9) Manoj, D.; Rajendran, S.; Hoang, T. K. A.; Ansar, S.; Joo, S.-W.; Vasseghian, Y.; Soto-Moscoso, M. In-Situ Growth of 3D Cu-MOF on 1D Halloysite Nanotubes/Reduced Graphene Oxide Nanocomposite for Simultaneous Sensing of Dopamine and Paracetamol. *Journal of Industrial and Engineering Chemistry* **2022**, *112*, 287–295. https://doi.org/10.1016/j.jiec.2022.05.022.
